# Comparative Efficacy and Safety of Neoadjuvant Immunotherapy with Nivolumab vs. Pembrolizumab in Resectable Non-Small Cell Lung Cancer: A Systematic Review

**DOI:** 10.3390/curroncol31100469

**Published:** 2024-10-18

**Authors:** Anastasia Papaporfyriou, Konstantinos Bartziokas, Ioulianos Apessos, Jan Mueller, Vasileios Leivaditis, Efstratios Koletsis, Konstantinos Grapatsas

**Affiliations:** 1 Department of Pulmonology, Internal Medicine II, Medical University of Vienna, 1090 Vienna, Austria; jan.mueller@meduniwien.ac.at; 2 Independent Researcher, 42100 Trikala, Greece; bartziokas@gmail.com; 3 Department of Dentoalveolar Surgery, Implantology and Oral Radiology, School of Dentistry, Faculty of Health Sciences, Aristotle University of Thessaloniki, 54124 Thessaloniki, Greece; julianapessos@hotmail.com; 4 Department of Cardiothoracic and Vascular Surgery, WestpfalzKlinikum, 67655 Kaiserlautern, Germany; vnleivaditis@gmail.com; 5 Department of Cardiothoracic Surgery, Patras University Hospital, 26504 Patras, Greece; ekoletsis@hotmail.com; 6 Department of Thoracic Surgery, West German Cancer Center, University Medical Center Essen-Ruhrlandklinik, University Duisburg-Essen, 45239 Essen, Germany; grapatsaskostas@gmail.com

**Keywords:** nivolumab, pembrolizumab, neoadjuvant immunotherapy, non-small cell lung cancer, early-stage non-small cell lung cancer, preoperative therapy, treatment efficacy, safety profiles

## Abstract

Non-small cell lung cancer (NSCLC) remains a leading cause of cancer-related mortality worldwide. Immunotherapy has emerged as a promising treatment option due to its favorable toxicity profile. However, selecting the most appropriate immunotherapeutic agent for neoadjuvant use—aimed at curative intent in early-stage NSCLC—based on efficacy and safety remains a critical question. This review aims to compare the efficacy and safety profiles of nivolumab and pembrolizumab when used as neoadjuvant treatments in NSCLC. A systematic review was conducted across PubMed, Scopus, Wiley Online Library, ProQuest Dissertations and Theses Global, and Google Scholar, utilizing the search terms “Nivolumab OR Pembrolizumab AND Neoadjuvant Immunotherapy AND non-small cell lung cancer.” Out of 1444 retrieved studies, 4 retrospective studies met the inclusion criteria by providing comparative data on nivolumab and pembrolizumab within the same study cohorts. Despite the critical risk of bias and the evidence quality ranging from moderate to very low across these studies, both nivolumab and pembrolizumab demonstrated efficacy rates exceeding 30% and maintained favorable safety profiles. There is no observed superiority between nivolumab and pembrolizumab in terms of efficacy and safety for the neoadjuvant treatment of early-stage NSCLC.

## 1. Introduction

Non-small cell lung cancer (NSCLC) represents 80–85% of all lung cancers globally and remains the principal cause of cancer-related deaths according to the 2020 global cancer statistics [1]. Improving patients’ long-term survival should be the top priority. It is thought that neoadjuvant treatment can improve patients’ longevity by reducing staging and raising the R0 resection rate [2]. In patients with stage IB–IIIA NSCLC, recent evidence has shown that neoadjuvant immunotherapy and targeted treatments can enhance overall survival, progression-free survival, or event-free survival [3,4]. More tools are needed to create a personalized treatment plan for individuals receiving composite treatment modalities.

**Figure 1:**
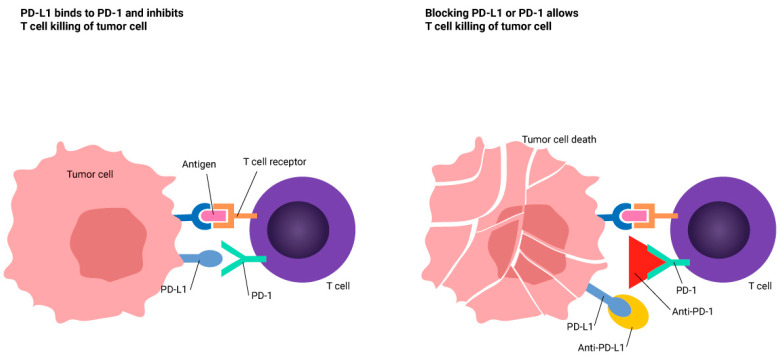
The interaction mechanism of anti-PD-1 with its targets.

The treatment landscape for NSCLC has shifted dramatically, starting with the discovery of oncogenic mutations, such as the EGFR mutation in 2004 [5], which significantly improved patient survival and quality of life. Another major advancement is immunotherapy [6], notably the use of PD-1 and CTLA-4 immune checkpoint inhibitors (ICIs), which has been transformative since 2013, altering the treatment landscape not just for NSCLC, but also for small cell lung carcinoma [7,8]. Monoclonal antibodies targeting the programmed cell death protein 1/programmed death-ligand 1 (PD-1/PD-L1) axis are approved as both first- and second-line treatments for advanced NSCLC [9]. Following the success of immune checkpoint inhibitors (ICIs) in metastatic settings, there has been burgeoning research into their neoadjuvant use [10,11,12,13]. Although a reliable biomarker predicting the effectiveness of neoadjuvant PD-1/PD-L1 blockades is yet to be found, a lot of interest has gained around the expression of PD-L1 (see Figure 1) following the studies showing responses to ICIs according to their expression in advanced NSCLC [14]

Preclinical theories posit that exposing immune cells to tumor antigens pre-surgery could act as a “vaccine”, thus enhancing immune response, as evidenced by improved pathological responses and tumor downstaging compared to neoadjuvant chemotherapy [15,16]. Recent meta-analyses suggest that, in combination with chemotherapy, anti-PD-1 therapies are superior in efficacy and safety to anti-PD-L1 therapies [17], echoing earlier findings in non-resectable NSCLC that demonstrated a 32% reduction in the risk of death with anti-PD-1 therapies compared to anti-PD-L1 therapies [18,19]. Given these insights, this review critically examines the clinical benefits of two anti-PD-1 immunotherapies, nivolumab and pembrolizumab, in the treatment of NSCLC. We systematically reviewed clinical trial data, focusing on the perioperative use of these agents in patients with potentially curable NSCLC, analyzing efficacy-related endpoints including major pathological response (MPR) and complete pathological response (pCR), which are currently utilized as surrogate endpoints for predicting survival benefits in clinical trials on neoadjuvant chemotherapy in stages I–III resectable NSCLC [20].

## 2. Materials and Methods

### 2.1. Protocol and Registration

The protocol for this systematic review was registered with the International Prospective Register of Systematic Reviews (PROSPERO) under the registration ID CRD42024510972. The guidance for our systematic review was the “Cochrane Handbook for systematic reviews of Interventions” [21]. The review was conducted and reported in adherence to the Preferred Reporting Items for Systematic Reviews and Meta-Analyses (PRISMA) criteria [22].

### 2.2. Eligibility Criteria

#### Inclusion and Exclusion Criteria

Eligible studies involved human patients diagnosed with NSCLC. Exclusion criteria were as follows:

(a) Types of publications: Reviews, meta-analyses, abstracts, letters, editorials, conference proceedings, laboratory studies, and animal studies were excluded.
(b) Language restrictions: Non-English studies were excluded.
(c) Cancer type: Studies focusing on cancers other than NSCLC were excluded.
(d) Therapy use: Studies not evaluating immunotherapy as a neoadjuvant treatment were excluded.
(e) Specificity of treatment: Studies that did not assess nivolumab or pembrolizumab as neoadjuvant therapies were excluded.

### 2.3. Information Sources and Search Strategy

The literature search was conducted independently by two authors (AP and IA) using databases such as PubMed, Scopus, Wiley Online Library, ProQuest Dissertations and Theses Global, and Google Scholar. The last search was conducted on 5 August 2024. Search terms included “Nivolumab OR Pembrolizumab AND as Neoadjuvant Immunotherapy AND non-small cell lung cancer.” Search filters applied included English language for Scopus, publication years from 2013 to 2024 for Wiley, and dissertations and theses for ProQuest (See Table S1 in the Supplementary Material for the search strategy in detail).

### 2.4. Definitions, Interventions, and Outcome Measures

Neoadjuvant immunotherapy refers to the administration of immunotherapy before surgical intervention in NSCLC. Treatment efficacy was assessed using major pathological response (defined as ≤10% viable tumor cells remaining after surgery) and complete pathological response (defined as 0% viable tumor cells remaining). The primary endpoint was to compare the efficacy of nivolumab versus pembrolizumab. Secondary endpoints included their efficacy relative to PD-L1 expression rates and safety profiles concerning surgical complications.

### 2.5. Data Management, Study Selection, and Data Extraction

Search results were managed using Rayyan 1.4.3 Version, a collaborative online software for systematic reviews [23]. After removing duplicates, titles and abstracts were screened by two independent reviewers (AP and KB). Discrepancies were resolved through discussion, or, if unresolved, escalated to full-text review. Detailed reasons for exclusion were recorded, and in cases of persistent disagreement, a third reviewer adjudicated. Data were extracted using a custom sheet, including principal author, publication year, study design, population, and outcomes comparing the efficacy of pembrolizumab versus nivolumab.

### 2.6. Risk of Bias Assessment

The risk of bias of non-randomized studies was assessed with the Cochrane risk of bias tool for non-randomized studies of intervention (ROBINS-I tool, Version 2016), which assesses the following 7 domains of bias: (i) confounding, (ii) selection of participants into the study, (iii) classification of interventions, (iv) deviations from intended interventions, (v) missing data, (vi) measurement of outcomes, and (vii) selection of the reported result. Possible risk of bias judgments were: “low risk of bias”, “moderate risk of bias”, “serious risk of bias”, “critical risk of bias”, and “no information” [24].

### 2.7. Quality of Evidence across Studies

The overall quality of evidence (confidence in effect estimates) was assessed using the Grading of Recommendations Assessment, Development, and Evaluation (GRADE) working group methodology [25]. GRADEpro software Version 2024 [26] was used to assess the quality of evidence across the domains of risk of bias, consistency, directness, precision, and publication bias. Quality levels were classified as “high”, “moderate”, “low”, or “very low”, reflecting the likelihood that further research might alter the confidence in and the impact of the effect estimates.

## 3. Results

### 3.1. Study Selection and Characteristics

**Figure 2:**
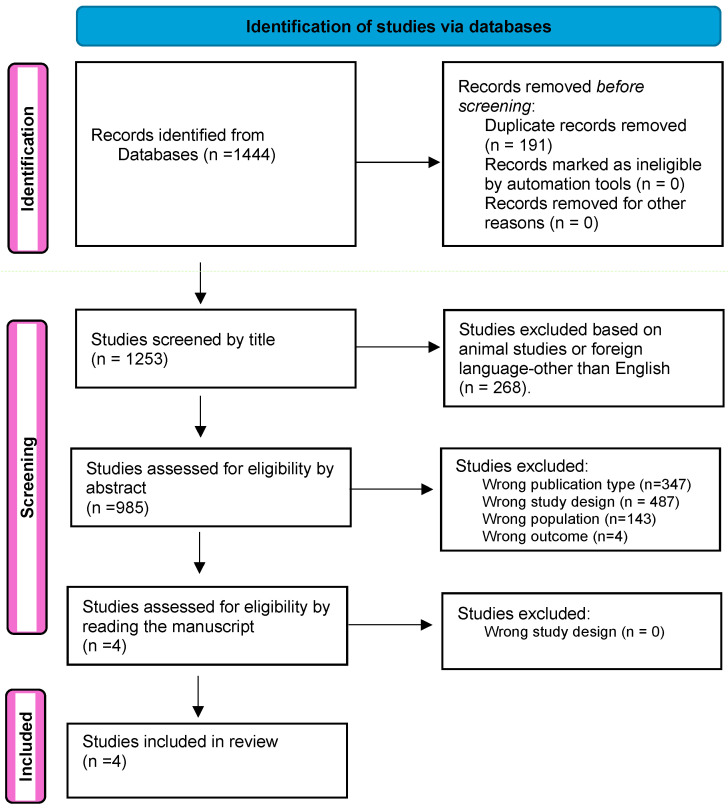
PRISMA flow diagram. This diagram illustrates the process of study selection and inclusion for the systematic review, detailing the number of studies identified, screened, deemed eligible, and included in the final analysis.

The search strategy and selection process for eligible studies are summarized in the PRISMA flow diagram (Figure 2). From the initial 1444 studies retrieved, only 4 met the inclusion criteria and were included in our analysis. These studies provided comparative data on both nivolumab and pembrolizumab within the same study samples. The details of the included studies are presented in Table 1.

### 3.2. Risk of Bias within Studies

**Figure 3:**
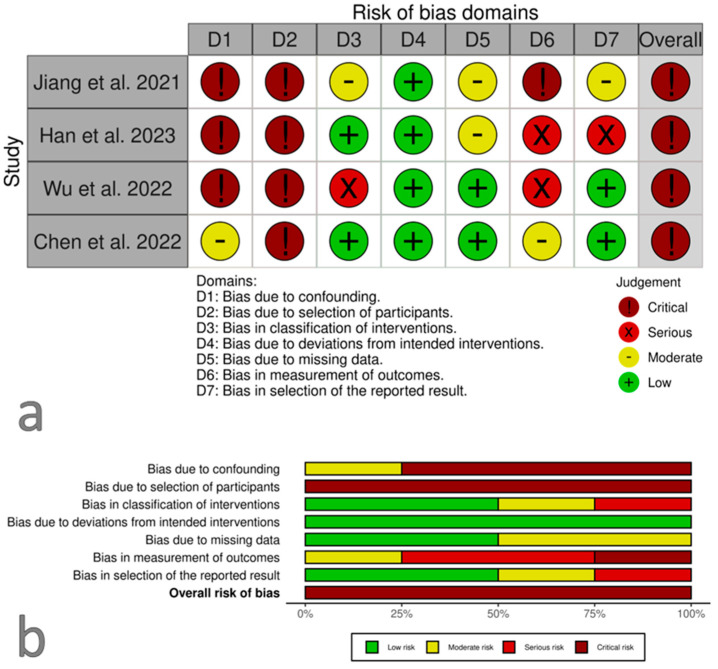
Risk of bias summary plots using the ROBINS-I tool. Panel (**a**) displays the risk of bias evaluated for each individual study included in the systematic review [27,28,29,30]. Panel (**b**) illustrates the risk of bias according to each domain assessed using the ROBINS-I tool, providing a comprehensive view of the potential biases affecting the study results.

The risk of bias for all included studies was judged as critical. Summary plots of the risk of bias are depicted in Figure 3. The risk of bias was rated as critical in all included studies. This was mainly due to the lack of adjustment for confounding factors and selection bias due to the retrospective design. The factors predicting the outcome of interest, in particular MPR or cPR, cannot completely exclude the possibility that a patient received one or the other immunotherapy.

### 3.3. Risk of Bias across Studies

The quality of evidence for all outcomes was assessed as ranging from moderate to very low, with detailed results presented in Table 2. The main reasons for downgrading the quality of evidence pertained to: (1) the inclusion of non-randomized studies with serious methodological issues, (2) the indirectness attributed to the presence of a different timeframe of immunotherapy received before radical surgery, whereas a direct comparison of pembrolizumab vs. nivolumab’s effectiveness was not the primary outcome of the included studies, and (3) the imprecision of estimates due to narrative synthesis. This means that further research with well-designed studies is very likely to have an important impact and is likely to change our current estimates of effect.

### 3.4. Results of Data Synthesis

Except for the study by Chen et al. [30], where a significant disparity in patient numbers was observed between the pembrolizumab (8 patients) and nivolumab (29 patients) groups, the remaining studies had a relatively equal distribution of patients across both treatment groups. Regarding our primary endpoint, the major pathological response (MPR) rates for pembrolizumab were 33% in studies by Jiang et al. [27] and Han et al. [28], and 31% and 50% in the studies by Wu et al. [29] and Chen et al. [30], respectively. For nivolumab, the MPR rates were 43%, 23%, and 72% in the studies by Jiang et al., Wu et al., and Chen et al., respectively. Complete pathological response (CPR) was reported in the studies by Han et al. and Wu et al., achieving rates of 50% and 40% in the pembrolizumab group and 100% and 32% in the nivolumab group, respectively.

As a secondary outcome, PD-L1 expression levels greater than 1% were found in 43% and 50% of patients receiving pembrolizumab and nivolumab, respectively, in the study by Wu et al. In the studies by Jiang et al. [27] and Han et al. [28], PD-L1 expression greater than 1% was observed in 38% and 63% of all patients, respectively, while in the study by Chen et al. [30], all patients exhibited PD-L1 expression over 1%.

Regarding safety, the studies by Jiang et al. [27] and Wu et al. [29] noted no significant differences in mortality and major postoperative complications compared to studies involving adjuvant immunotherapy. Major complications from immunotherapy reported as grade 3 from the study of Wu et al. included neutropenia (4/42 and 3/34 for the pembrolizumab and nivolumab groups, respectively), increased aminotransferases (1/42 and 4/34), skin rash (3/42 and 1/34), anemia (3/42 and 2/34), hyponatremia (1/42 and 1/34), and urinary tract infections (0/42 and 1/34) [29].

### 3.5. Studies Focusing on Surgery

In only two studies were surgical procedures analyzed in depth [27,29]. These studies collectively included 107 patients. Video-assisted thoracic surgery (VATS) was performed in 56 patients (52.3%), with 48 undergoing uniportal VATS. Robotic-assisted thoracic surgery (RATS) was conducted in one patient, while the remainder underwent open surgical approaches. The most common procedure was lobectomy; however, sleeve lobectomy was performed in ten patients, and bilobectomy was necessary in one case. Pneumonectomy was required for complete tumor resection in five patients. Conversion from VATS to thoracotomy was required in only three cases.

Intraoperative data reported in the study by Jiang et al. [27] indicated a median operative time of 158 min (range: 77–279 min) and a median estimated blood loss of 200 mL (range: 50–1600 mL). Only two patients required blood transfusions. The median length of hospital stay post-surgery was 7 days (range: 2–29 days).

The most commonly encountered postoperative complications were prolonged air leaks and pneumonia [29]. However, no perioperative mortality was reported. Major postoperative complications, including hemothorax or pneumonia, were observed in 2 out of 20 patients, consistent with rates observed following conventional sleeve lobectomy. Other postoperative complications, such as air leaks (14/21), chylothorax (1/21), arrhythmias (3/21), and wound infections (1/21), occurred within expected rates for such procedures, even without the addition of neoadjuvant treatment [27]. No postoperative mortality was reported in either study.

## 4. Discussion

To the best of our knowledge, this systematic review is the first to compare the efficacy of PD-1 antibodies, pembrolizumab and nivolumab, as neoadjuvant therapies in NSCLC, with studies incorporating both regimens [17,31,32]. These agents are approved for pre-surgical treatment in patients with resectable NSCLC. Traditionally, overall survival is deemed the gold standard for evaluating NSCLC therapy effectiveness; however, the protracted time required to assess this endpoint has led to the adoption of intermediate measures such as MPR or CPR following surgery [31]. The correlation between these surrogate endpoints and long-term survival outcomes remains to be fully established, suggesting an area for future research.

In our review, MPR for pembrolizumab ranged from 31% to 50%, while for nivolumab, it was between 23% and 72%. Similarly, CPR for pembrolizumab was between 40% and 50%, and for nivolumab, it ranged from 32% to 100%. The broad variability, especially in the nivolumab group, is likely due to the small sample sizes in the studies examined. Mei et al.‘s meta-analysis supports nivolumab’s superior performance in achieving CPR post-surgery compared to pembrolizumab [17]. However, there is evidence that pembrolizumab combined with chemotherapy enhances the objective response rate, progression-free survival, and overall survival across all PD-L1 expression statuses, positioning it as a potentially more effective regimen when effective [32]. In cases where pembrolizumab combined with chemotherapy is ineffective, nivolumab appears to be a viable alternative.

The studies included in this review were specifically designed to minimize bias by including patients who were eligible for either therapy, rather than focusing exclusively on one, thereby reducing methodological biases. Despite this, the limited number of studies and their small sample sizes make it difficult to draw definitive conclusions.

In both the adjuvant and metastatic settings for NSCLC, PD-L1 expression is used as a biomarker to guide the selection of immunotherapy [33]. While some evidence points to PD-L1 as a predictor of treatment benefit in the neoadjuvant setting [3], the data are mixed, and in our review, for example, patients with PD-L1 status <1% also showed encouraging results regarding their pathological response after immunotherapy [27]. No consensus protocols have yet been established for routinely using PD-L1 status as a biomarker to determine eligibility for neoadjuvant chemoimmunotherapy.

Safety data from our review indicate that neoadjuvant immunochemotherapy is well tolerated, with no serious adverse events such as death or severe surgical complications reported. Common immune-related adverse events included pneumonitis, thyroid dysfunction, myelosuppression, and increased susceptibility to infections and skin rash, which, although potentially serious, are generally manageable with prompt and effective treatment [34,35]. Neoadjuvant immunochemotherapy in patients with NSCLC requires close monitoring and timely reporting of any symptoms to the healthcare practitioner.

Looking ahead, there is a critical need for larger multicenter trials that can provide more robust data to validate the efficacy and safety profiles of neoadjuvant immunotherapy. Further research should aim to clarify the relationship between surrogate endpoints like MPR and CPR and long-term survival, potentially establishing these measures as reliable predictors of overall survival. Additionally, exploring the genetic and molecular profiles of tumors might yield insights into which patients are most likely to benefit from specific immunotherapies, thereby personalizing treatment approaches. The development of protocols to better utilize biomarkers like PD-L1 in clinical decision-making remains an urgent requirement. Ongoing and future studies should focus on optimizing treatment sequences and combinations, particularly exploring the synergistic potential of combining immunotherapy with other modalities such as targeted therapy or radiation.

## 5. Limitations

The primary factor contributing to the downgrading of the quality of evidence in this review stems from the inclusion of non-randomized studies that possess significant methodological shortcomings. These issues include lack of blinding, retrospective design, low number of patients, and the heterogeneity in patient populations regarding cancer type and stage. Such methodological concerns underscore the necessity for further research through well-designed, prospective, randomized controlled trials. Conducting these studies is essential to potentially alter our current understanding and improve the reliability of the effect estimates for neoadjuvant immunotherapy in NSCLC.

## 6. Conclusions

This systematic review has confirmed the safety of neoadjuvant immunotherapy in patients with resectable NSCLC. Patients with locally advanced tumors are often ideal candidates for neoadjuvant treatments, which can potentially reduce tumor size and subsequently lessen the extent of the lung resection required. The efficacy of neoadjuvant immunotherapy should be evaluated by a multidisciplinary tumor board to ensure comprehensive patient care. In cases with technical challenges related to tumor resection, minimally invasive techniques proved beneficial and were successfully implemented without leading to significant postoperative complications. There were no instances of excessive or unexpected adverse effects, and, notably, no postoperative mortality was reported. These findings underline the potential of neoadjuvant immunotherapy not only to facilitate surgical outcomes, but also to maintain a high safety profile, even in complex clinical scenarios. For future practice, it is crucial to continue monitoring the long-term outcomes and further investigate the impact of neoadjuvant immunotherapy on patient survival and quality of life. This will help to solidify its role in the standard treatment regimen for NSCLC.

## Figures and Tables

**Table 1:** Summary of the key characteristics of included studies in the systematic review.

Authors, Ref.	Methods	Participants	Interventions	Primary Outcomes	Outcomes for the Efficacy of Nivolumab vs. Pembrolizumab	Secondary Outcomes - PD-L1 Staining
Long Jiang, et al, 2021 [27]	RCS. pCR was defined as 0% viable tumor cells in residual tumor, while major pathological response as 10% remaining.	31 NSCLC Patients stage IIA (1), IIB (4), IIIA (16), IIIB (10)	Neoadjuvant chemoimmunotherapy (27) or immunotherapy alone (4) with Pembrolizumab (15) or Nivolumab (16).	Analysis of surgical perspective outcome data after neoadjuvant immunotherapy followed by surgery for resectable NSCLC.	Pembrolizumab MPR (5/15 = 33%). Nivolumab MPR (7/16 = 43%).	PD-L1 < 1%: 10/31. PD-L1 > 1%: 12/31. unknown PD-L1 status: 9/31. MPR in PD-L1 < 1%: 5/22. MPR in PD-L1 > 1%: 3/22.
Rui Han, et al., 2023 [28]	RCS. Pathological assessment according to the multidisciplinary recommendations of the IASLC (MPR < 10% viable tumor cells, pCR: 0%).	29 NSCLC Patients stage III (23), stage IV (6)	Neoadjuvant Chemoimmunotherapy with Pembrolizumab (6), Nivolumab (5), Sentilimab and Tislelizumab (18).	Clinical features of patients with good pathological response to neoadjuvant chemoimmunotherapy and potential biomarkers that discriminate population sensitivity to this therapy.	Pembrolizumab cPR (3/6 = 50%) &MPR (2/6 = 33%). Nivolumab cPR (5/5 = 100%).	Treated patients had either > 1% (7/11) or unknown (4/11) PD-L1 status.
Junqi Wu, et al., 2022 [29]	RCS. MPR: no more than 10% viable tumor cells, pCR: the absence of viable tumor cells in all slides. In addition, tumor bed without any characteristic of treatment related response was classified as NR. The presence of treatment-associated necrosis or fibrotic tissue while vital tumors cells > 10% was labeled PR.	76 Patients with NSCLC: Stage IB:1, Stage IIB:5, Stage IIIA: 41, Stage IIIB: 29.	Chemoimmunotherapy with Nivolumab: 34. Chemoimmunotherapy with Pembrolizumab: 42.	The feasibility, safety, and antitumor activity of pembrolizumab or nivolumab plus chemotherapy in treatment-naive and driver mutation negative patients with potentially resectable NSCLC.	Pembrolizumab MPR: 13/42 = 31%, pCR: 17/42 = 40%, PR: 8/42, NR: 4/42. Nivolumab MPR: 8/34 = 23%, pCR:11/34 = 32%, PR: 13/34, NR: 2/34.	PD-L1 < 1% with Pembrolizumab: 19/42. PD-L1 > 1% with Pembrolizumab: 18/42 = 43%. PD-L1 < 1% with Nivolumab: 11/34. PD-L1 > 1% with Nivolumab: 17/34 = 50%.
Zhi-Yong Chen, et al., 2022 [30]	RCS. MPR is defined as ≤ 10% of the viable tumor.	44 Patients with NSCLC II-III Stage	Immunotherapy +/− chemotherapy: 29 Pat. with Nivolumab, 8 Pat. with Pembrolizumab.	The association of the dynamic changes in PET/CT with MPR in patients receiving different preoperative immunotherapies.	21/29 = 72% MPR with Nivolumab. 4/8 = 50% MPR with Pembrolizumab.	All patients had > 1% PD-L1 status.

**Table 2:** A comprehensive summary of the findings, assessing the quality of evidence and the strength of recommendations for each outcome, based on the GRADE methodology.

Pembrolizumab Compared to Nivolumab for NSCLC			
**Patient or population:** Patients with NSCLC **Setting**: Hospitals and University Hospitals (China) **Intervention**: Pembrolizumab **Comparison**: Nivolumab			
Outcome № of participants (studies)	Impact	Certainty	What Happens with immunotherapy
Efficacy of Immunotherapy with pembrolizumb vs. nivolumab assessed with: MPR (No more than 10% viable tumor cells in the primary tumor bed), CPR (Absence of viable tumor cells in the primary tumor bed) № of participants: 155 (4 observational studies)	Out of 71 patients that received pembrolizumab, 29 presented MPR and 25 presented CPR. Out of 84 patients that received nivolumab, 38 presented MPR and 23 presented CPR	⨁◯◯◯ VERY LOW ^a,b,c^	Too heterogenous response to synthesize across studies.

**Explanations:** a. Downgraded by two levels because all included studies are non-randomized with critical risk of bias. b. Indirectness attributed to the presence of a different time frame of immunotherapy received before radical surgery, whereas direct comparison of pembrolizumab vs. nivolumab’s effectiveness was not the primary outcome of the included studies. c. Narrative synthesis was conducted. Estimates are not precise.

## Data Availability

The authors confirm that the data supporting this study’s results are available within the present review.

## Supplementary Materials

The following supporting information can be downloaded at: https://www.mdpi.com/article/10.3390/curroncol31100469/s1, Table S1: Search strategy for the databases in our systematic review.
