# Supplementary material for: Comparative Efficacy and Safety of Neoadjuvant Immunotherapy with Nivolumab vs. Pembrolizumab in Resectable Non-Small Cell Lung Cancer: A Systematic Review
Source: Curr Oncol. 2024 Oct 18;31(10):6289–99. doi: 10.3390/curroncol31100469 (PMC11506529; doi:10.3390/curroncol31100469)
Supplement: Supplementary file 1 [file curroncol-31-00469-s001.zip › curroncol-3189649-supplementary.pdf]

DATABASE	SEARCH STRATEGY	RESULTS
PUBMED	Advanced Search until 05.08.24: Nivolumab OR Pembrolizumab AND as Neoadjuvant Immunotherapy AND non-small cell lung cancer	101 Results
SCOPUS	Normal Search until 05.08.24: Nivolumab OR Pembrolizumab AND as Neoadjuvant Immunotherapy AND non-small cell lung cancer	246 Results
WILLEY	Advanced Search until 05.08.24: Nivolumab OR Pembrolizumab AND as Neoadjuvant Immunotherapy AND non-small cell lung cancer	591 Results
PROQUEST	Advanced Search until 05.08.24: Nivolumab OR Pembrolizumab AND as Neoadjuvant Immunotherapy AND non-small cell lung cancer	431 Results
GOOGLE SCHOLAR	Search strategy all in title until 05.08.24: Nivolumab OR Pembrolizumab AND as Neoadjuvant Immunotherapy AND non-small cell lung cancer	75 Results

Supplementary Table S1: Search strategy for the databases in our systematic review
